# Patient-nominated supporters as facilitators for engagement in HIV care in a referral hospital in Peru: A retrospective cohort study

**DOI:** 10.1371/journal.pone.0195389

**Published:** 2018-04-04

**Authors:** Mateo Prochazka, Larissa Otero, Kelika A. Konda, Elsa González-Lagos, Juan Echevarría, Eduardo Gotuzzo

**Affiliations:** 1 Instituto de Medicina Tropical Alexander von Humboldt, Universidad Peruana Cayetano Heredia, Lima, Peru; 2 Carlos Vidal Layseca School of Public Health and Management, Universidad Peruana Cayetano Heredia, Lima, Peru; 3 Alberto Hurtado School of Medicine, Universidad Peruana Cayetano Heredia, Lima, Peru; 4 Department of Medicine, Division of Infectious Diseases and Center for World Health, David Geffen School of Medicine at University of California at Los Angeles, Los Angeles, California, United States of America; 5 Department of Infectious, Tropical and Dermatological Diseases, Hospital Cayetano Heredia, Ministry of Health, Lima, Peru; The Ohio State University, UNITED STATES

## Abstract

Patient-nominated supporters can potentially improve the continuum of HIV care. We retrospectively determined factors associated with having a patient-nominated supporter among people living with HIV (PLWH), and its association with retention in care and viral suppression. We analysed registries of adults evaluated by social workers (n = 1345) at a referral hospital in Peru between 2011–2014. Nondisclosure of HIV status was associated with lacking supporters (aPR: 5.41, 95% CI: 3.83–7.64). Retention in care was 76.4% and 34.2% after one and two years of enrolment, respectively. PLWH with supporters were more likely to be retained in care after two years (aRR = 1.36, 95% CI: 1.02–1.81), but not after one year (aRR = 1.10, 95% CI: 0.98–1.23) compared to PLWH without supporters. Having supporters who were parents or friends was associated with an increased probability of being retained in care after one and two years of enrolment. Viral suppression after one year of enrolment was 58.7%. Having a supporter was not associated with viral suppression (aRR = 1.18, 95% CI: 0.99–1.41), but PLWH with supporters were more likely to have viral load measurements (p = 0.005). Patient-nominated supporters appear beneficial for engagement in HIV care; these benefits may be related to the nature of their relationship with PLWH.

## Introduction

People living with HIV (PLWH) can thrive through combined antiretroviral treatment (ART) and engagement with multidisciplinary care [1]. Poor adherence to treatment and clinical appointments results in disease progression, detectable viral load, increased transmission to others, and death [1–3]. To manifest the individual and public health benefits of HIV treatment, health systems should actively aim to improve retention in care [4]. In Latin America, ART delivery has dramatically increased in the past decade, but the current proportion of PLWH retained in care is estimated to be low, ranging from 64 to 77% [5–7]. Hospital-based cohorts in Peru report that less than 60% of patients are retained in care one year after linkage, with further reductions in long-term retention [8]. This is well below the 90-90-90 target needed for HIV epidemic control [9,10].

Socio-ecological frameworks highlight the role of interpersonal factors in shaping outcomes such as retention in care [11]. Social support is the perception and actuality of being cared for, having assistance from others, and being part of a supportive social network [12]. Perceived social support has been identified as having direct effects on physical and mental health among PLWH [13], and as protective against the negative effects of stigma on HIV symptoms [14]. A qualitative study published in 2010 described interpersonal relationships and support from others as facilitators for ART adherence in Peru [15], which has also been observed in other settings [16,17]. Peer, buddy or supporter-based strategies are a tangible representation of social support, and have been associated with decreased felt stigma scores and improved treatment outcomes in South Africa [18,19]. However, there is still uncertainty on whether these strategies are efficacious to improve retention in care [4]. The provision of instrumental support by others might address powerful health-system barriers, such as difficulties obtaining appointments or accessing health care services periodically [20,21]. Moreover, patient-nominated supporters may facilitate retention by combating stigma, restoring hope and influencing health-related decision-making [21–23].

In Peru, all PLWH who link to care at a government hospital are asked to nominate a supporter [24]. These patient-nominated supporters are expected to provide companionship and instrumental aid throughout the continuum of HIV care. They may substitute PLWH in ART pick-up appointments and schedule clinical visits for the PLWH they support, potentially addressing barriers such as strict work schedules or other competing responsibilities [25]. However, some PLWH may not be able to nominate supporters and may subsequently be deprived of the supporter’s facilitating role. We conducted this cohort study of PLWH with two objectives. First, to determine the characteristics associated with not having a patient-nominated supporter; and second, to study the association between having patient-nominated supporters and follow-up outcomes including retention in care and viral suppression.

## Methods

### Study design and participants

This retrospective cohort study analysed patient registries from a referral Ministry of Health hospital in Lima, Peru. We included all adult PLWH (≥18 years old) who enrolled in the National HIV Program (NHP) of this hospital between Jan 1^st^ 2011 and Dec 31^st^ 2014 and linked to medical care. We defined “linked” as having an outpatient medical evaluation within 90 days after an enrolment interview with NHP nurses [26]. We excluded PLWH who were not evaluated by social workers as supporters are nominated during these evaluations. Follow-up included attendance to medical visits and viral load counts from enrolment until Dec 31^st^ 2016.

### Study setting

During NHP enrolment interviews, nurses refer PLWH to CD4 and viral load (VL) testing, to schedule a medical evaluation at the infectious diseases (ID) clinic, and an evaluation with social workers. Blood samples taken on site are processed at a national central laboratory and results are available within a month. PLWH are asked to attend social work evaluations with a close person who can act as a patient-nominated supporter; such evaluations are particularly encouraged before ART initiation. During the appointment, PLWH are asked to formally register up to two supporters. If PLWH are unable to nominate supporters, social workers may enlist NHP peer counsellors as supporters. Government insurance usually covers for all care expenses. Free ART is offered based on national treatment guidelines, which were a CD4 count below 200 cells/mm^3^ before 2012, below 350 cells/mm^3^ from 2013 to 2014 and below 500 cells/mm^3^ after 2015 [24]. AIDS-defining diseases, co-infection with Hepatitis B Virus, tuberculosis, or pregnancy are also indications for ART initiation regardless of CD4 count [24].

### Data management

Data management and analysis was performed using Stata 13.0. Data for this study was obtained from three sources of routine data. NHP nurses register demographics, dates of diagnosis, CD4 counts, viral loads, World Health Organization (WHO) clinical staging, ART pick-up dates, and dates of death in the “NHP database”. PLWH socio demographics and patient-nominated supporter information are entered into the “Social work database”. Finally, data on all outpatient visits attended are entered in the “ID outpatient database”. We matched registries from the Social work database and the ID outpatient database to the NHP database, as the NHP database included all PLWH identified during the study period. We used multiple combinations of compound identifiers in merging algorithms. We combined the resulting datasets, eliminated duplicates, and manually addressed unmatched registries to verify the absence of homologous registries. Two researchers (MP and EGL) merged the databases separately. Discrepancies were solved upon verification. All identifiers were removed from the final study database. Prior to analysis, we compared PLWH evaluated by a social worker with those who were not to assess for selection bias.

Having a supporter was defined as having a person named in one of the patient-nominated supporter fields in the Social work database. NHP counsellors were not considered as “supporters” because they were not patient nominated. The kinship with the PLWH was classified as partner, parent, friend, sibling, second-degree relative, or offspring. Disclosure to family members was classified as nondisclosure or disclosure to ≥1 family member, excluding partners. PLWH socio-demographics included sex at birth (male, female), age (categorized as <32 years, ≥32 years based on a median split), self-reported behaviour as men who have sex with men (MSM, non-MSM, women), place of birth (Lima, other), unemployment (yes, no), educational level attained (primary or less, secondary, or higher), having a domestic partner (yes, no), receiving economic support from family (yes, no), and lack of all housing services including water, sewage and electric power (yes, no). Other variables included time with an HIV-positive diagnosis at enrolment (<30 days, 30–365 days, >365 days), first CD4 count (cells/mm^3^), and AIDS stage at enrolment, defined as having WHO clinical stage IV and/or CD4 count <200 cells/mm^3^ (yes, no) [27]. ART initiation was defined as the delivery of three or more antiretroviral drugs following medical prescription [24]. We defined known death by the registry of mortality in the NHP database.

### Statistical analysis

First, we sought to determine the characteristics associated with not having a patient-nominated supporter. We compared the demographics of PLWH that had a patient-nominated supporter to those who did not using Chi-square test, Fisher’s exact test, Student’s t test, and Wilcoxon rank-sum test depending on the variables type and distribution. We modelled not having a patient-nominated supporter using Poisson regression with robust variance to estimate prevalence ratios (PR) [28]. Having a supporter was used as a reference category. The modelling approach was exploratory and data-driven. We used manual forward selection to yield a final model with minimally sufficient adjustment; variables were selected based on bivariate p-value (significance considered at <0.200) including those with lowest p-value first. Variables remained in the adjusted model if they were associated with not having supporters (p<0.05) or if they produced a >10% change in any estimates [29,30].

Second, we studied the association between having a patient-nominated supporter and three follow-up outcomes: retention in care after one year, retention in care after two years, and viral suppression. We defined “retention” as having ≥2 medical provider visits in the year following enrolment, >90 days apart [26]. For the two-year retention metric, we classified the PLWH as retained if they met the metric in both years consecutively [8]. We defined “suppression” as achieving an HIV-1 RNA <200 copies/mL at the last measurement within the first year of enrolment [31]. Individuals with a missing viral load measurement were classified as “not suppressed”. In a sensitivity analysis, we excluded individuals with missing viral load measurements from the viral suppression denominator.

We modelled the association between having a patient-nominated supporter and outcomes using Poisson regression with robust variance to estimate risk ratios (RR) [32]. For each outcome we performed two models: 1) having a patient-nominated supporter + confounders, including disease severity (AIDS) and time from diagnosis [33,34]; 2) kinship of the patient-nominated supporter + confounders, to assess for differential effects in outcomes by kinship. PLWH with two patient-nominated supporters were excluded from model 2 as dyads of supporters may outweigh individual effects. All models were adjusted for sex, age, and sexual behaviour [29]. For all our regression models, missing value analysis yielded a missing completely at random (MCAR) pattern. Observations with missing values for any of the included variables were excluded from the final model.

### Ethical considerations

The Institutional Review Boards of Universidad Peruana Cayetano Heredia and Hospital Cayetano Heredia approved the study protocol and granted a waiver for informed consent. Data were collected within the standard of care and analysed retrospectively.

## Results

### Study population

We found 1932 PLWH who linked to care. Of these, 1345 (69.6%) were evaluated by a social worker. PLWH evaluated by social workers were more likely to initiate ART (p<0.001) and to be retained in care after one and two years (p<0.001 for each outcome). The median time between enrolment and social worker evaluation was 17 days (interquartile range (IQR): 6–37). Table 1 summarizes characteristics and follow-up outcomes of PLWH included in this study.

**Table 1:** Characteristics and outcomes of people living with HIV who were evaluated by a social worker, 2011–2014.

Characteristics	N (%) (n = 1345)
Sex	
Female	316 (23.5)
Male	1029 (76.5)
Age (years)^a^	32.5 (26.4–41.6)
Sexual behavior	
Heterosexual	750 (55.8)
MSM	595 (44.2)
CD4 count (cells/mL)^a^	232 (101–399)
AIDS at baseline	
Yes	704 (52.8)
No	630 (47.2)
Time since diagnosis (days)^a^	29 (6–219)
Education	
Primary (1–6 years) or less	142 (11.1)
Secondary (7–11 years)	608 (47.4)
Higher (Technical or University)	534 (41.6)
Disclosure to family members	
Disclosure to one or more family members	1069 (82.7)
Nondisclosure	223 (17.3)
Domestic partnership	
Yes	359 (28.2)
No	914 (71.8)
Patient-nominated supporter	
Yes	1202 (89.4)
No	143 (10.6)
**Follow-up outcomes**	
Initiated ART	
Yes	1193 (88.7)
No	152 (11.3)
Time to ART initiation (days)^a^	43 (23–90)
Retention in care (1 year)	
Retained	1028 (76.4)
Not retained	317 (23.6)
Retention in care (2 year)	
Retained	460 (34.2)
Not retained	885 (65.8)
Viral suppression (1 year)^b^	
Suppressed	790 (58.7)
Not suppressed	555 (41.3)
Known death	
Yes	71 (5.3)
No	1274 (94.7)

ART = Antiretroviral treatment, MSM = Men who have sex with men, VL = Viral load

^a^Median (interquartile range)

^b^HIV RNA VL ≤200 copies/mL at the last measurement in the first year after enrolment

### Patient-nominated supporters

Among 1345 included PLWH, 1202 (89.4%) had supporters: 1138 (84.6%) nominated one supporter and 64 (4.8%) nominated two supporters. Patient-nominated supporters were parents (26.7%), siblings (22.5%), partners (20.7%), friends (14.5%), second-degree relatives (11.2%), or offspring (4.4%) of the PLWH. Supporters were mostly female (62.4%).

Supporter characteristics varied according to PLWH demographics. The frequency of female supporters was similar in women and MSM (55.8 and 57.1% respectively), but higher among non-MSM (74.2%, p<0.001). Women and non-MSM PLWH had higher frequency of partners as supporters compared with MSM (27.0% vs. 12.4%, p<0.001), while friends as supporters were more frequent among MSM compared with women and non-MSM (25.9% vs. 5.8%, p<0.001). PLWH aged 18–32 had higher frequency of parents as supporters than older PLWH (37.4% vs. 16.7%, p<0.001). Among PLWH who did not disclose their HIV status to family, supporters were mostly friends (62.2%) or partners (32.2%), but not relatives.

Table 2 compares PLWH with and without nominated supporters. Nondisclosure of HIV status to family members, not having a domestic partner, being born out of Lima, being older than 32 years or being diagnosed for more than 365 days at enrolment were all independently associated with not having a supporter. Unemployment, lack of house services and economic support from family were not included in the final model as they did not meet criteria to remain in the model. Sexual behaviour was included in the final model as it shifted the association between having a domestic partner and having a supporter by more than 10%.

**Table 2:** Factors associated with not having a patient-nominated supporter among people living with HIV who were evaluated by a social worker, 2011–2014.

Characteristics	Patient-nominated supporter (n = 1202)	No patient-nominated supporter (n = 143)	PR (95% CI)	aPR^a^ (95% CI)
Sex				
Female	288 (91.1)	28 (8.9)	Ref.	-
Male	914 (88.8)	115 (11.2)	1.26 (0.85–1.87)	-
Age				
18–32 years old	585 (91.3)	56 (8.7)	Ref.	Ref.
>32 years old	617 (87.6)	87 (12.4)	1.41 (1.03–1.94)	1.59 (1.14–2.22)
Sexual behavior				
Heterosexual	680 (90.7)	70 (9.33)	Ref.	Ref.
MSM	522 (87.7)	73 (12.3)	1.31 (0.96–1.79)	0.81 (0.56–1.16)
AIDS at baseline				
Yes	636 (90.3)	68 (9.7)	0.82 (0.60–1.12)	-
No	556 (88.2)	74 (11.8)	Ref.	-
Time since diagnosis (days)				
<30	588 (91.3)	56 (8.7)	Ref.	Ref.
30–365	331 (90.0)	37 (10.0)	1.15 (0.78–1.72)	1.27 (0.87–1.88)
>365	251 (85.1)	44 (14.9)	1.72 (1.18–2.48)	1.79 (1.23–2.61)
Place of Birth				
Lima Region	749 (91.0)	74 (9.0)	Ref.	Ref.
Elsewhere	452 (86.8)	69 (13.2)	1.47 (1.08–2.00)	1.40 (1.01–1.92)
Unemployment				
Yes	543 (92.2)	46 (7.8)	1.63 (1.16–2.28)	-
No	630 (87.3)	92 (12.7)	Ref.	-
Education level attained				
Primary (1–6) or less	130 (91.6)	12 (8.4)	0.90 (0.50–1.63)	-
Secondary (7–11)	551 (90.6)	57 (9.4)	Ref.	-
Higher (Technical or University)	470 (88.0)	64 (12.0)	1.28 (0.91–1.79)	-
Disclosure to family				
Disclosure to one or more family members	1010 (94.5)	59 (5.5)	Ref.	Ref.
Nondisclosure	149 (66.8)	74 (33.2)	6.01 (4.41–8.20)	5.41 (3.83–7.64)
Domestic partnership				
Yes	339 (94.4)	20 (5.6)	Ref.	Ref.
No	800 (87.5)	114 (12.5)	2.24 (1.41–3.54)	1.92 (1.13–3.24)
Economic support from family				
Yes	336 (92.8)	26 (7.2)	Ref.	-
No	865 (88.1)	117 (11.9)	1.66 (1.10–2.49)	-
Lack of house services				
Yes	65 (83.3)	13 (16.7)	1.62 (0.96–2.74)	-
No	1136 (89.7)	130 (10.3)	Ref.	-

MSM = Men who have sex with men, PR = Prevalence Ratio, CI = Confidence Interval, aPR = adjusted Prevalence Ratio

^a^Model adjusted for the variables shown in the table.

### Retention in care

Tables 3 and 4 show the association between having a patient-nominated supporter and retention in care after one and two years, respectively. Our adjusted models showed that PLWH who had supporters were more likely to be retained in care after two years, but not after one year adjusting for presenting with AIDS, time since diagnosis at enrolment, age, sex, and sexual behaviour. Having supporters who were parents or friends was associated with an increased probability of being retained in care after one and two years of enrolment, while having partners, second-degree relatives, siblings, or offspring as supporters was not associated with increased retention in care in any time period. Disclosure to family was not associated with retention in care after one or two years (p = 0.220 and p = 0.306, respectively).

**Table 3:** Associations between having patient-nominated supporters and being retained in care after one year among people living with HIV, 2011–2014.

Characteristics	Retained (n = 1028)	Not retained (n = 317)	RR (95% CI)	aRR (95% CI)
Patient-nominated supporter^a^				
Yes	928 (77.2)	274 (22.8)	1.10 (0.99–1.23)	1.10 (0.98–1.23)
No	100 (69.9)	43 (30.1)	Ref.	Ref.
Kinship of patient-nominated supporter^b^				
No supporter	100 (69.9)	43 (30.1)	Ref.	Ref.
Partner	179 (76.5)	55 (23.5)	1.09 (0.96–1.24)	1.06 (0.94–1.21)
Parent	243 (80.7)	58 (19.3)	1.15 (1.02–1.30)	1.17 (1.03–1.32)
Friend	132 (78.1)	37 (21.9)	1.12 (0.98–1.28)	1.15 (1.00–1.31)
Sibling	185 (74.5)	60 (24.5)	1.08 (0.95–1.23)	1.05 (0.92–1.20)
Second-degree relative	84 (72.4)	32 (27.6)	1.04 (0.89–1.21)	1.04 (0.89–1.21)
Offspring	36 (75.0)	12 (25.0)	1.07 (0.88–1.30)	0.98 (0.80–1.19)

All models were performed using Poisson regression with robust variances. RR = Risk Ratio, CI = Confidence Interval, aRR = adjusted Risk Ratio.

^a^Model includes the presence of a patient-nominated supporter adjusting for age, sex, sexual behavior, time since diagnosis, and AIDS at enrolment.

^b^Model includes the presence of a patient-nominated supporter disaggregated by kinship, adjusting for sex, sexual behavior, time since diagnosis, and AIDS at enrolment.

**Table 4:** Associations between having patient-nominated supporters and being retained in care after two years among people living with HIV, 2011–2014.

Characteristics	Retained (n = 460)	Not retained (n = 885)	RR (95% CI)	aRR (95% CI)
Patient-nominated supporter^a^				
Yes	423 (35.2)	779 (64.8)	1.36 (1.02–1.81)	1.36 (1.02–1.81)
No	37 (25.9)	106 (74.1)	Ref.	Ref.
Kinship of patient-nominated supporter^b^				
No supporter	37 (25.9)	106 (74.1)	Ref.	Ref.
Partner	71 (30.3)	163 (69.7)	1.17 (0.84–1.64)	1.16 (0.83–1.64)
Parent	114 (37.9)	187 (62.1)	1.45 (1.06–1.98)	1.56 (1.14–2.14)
Friend	70 (41.4)	99 (58.6)	1.62 (1.17–2.25)	1.59 (1.14–2.20)
Sibling	83 (33.9)	162 (66.1)	1.32 (0.96–1.83)	1.26 (0.91–1.75)
Second-degree relative	33 (28.5)	83 (71.6)	1.08 (0.73–1.61)	1.09 (0.73–1.62)
Offspring	19 (39.6)	29 (60.4)	1.60 (1.05–2.45)	1.21 (0.76–1.92)

All models were performed using Poisson regression with robust variances. RR = Risk Ratio, CI = Confidence Interval, aRR = adjusted Risk Ratio.

^a^Model includes the presence of a patient-nominated supporter adjusting for age, sex, sexual behavior, time since diagnosis, and AIDS at enrolment.

^b^Model includes the presence of a patient-nominated supporter disaggregated by kinship, adjusting for sex, sexual behavior, time since diagnosis, and AIDS at enrolment.

### Viral suppression

The median time between enrolment and the analysed VL measurement was 258 days (IQR: 219–309), this time was similar for PLWH with and without supporters (p = 0.880). A total of 995 (74.3%) PLWH had viral load measurements. PLWH who had patient-nominated supporters were more likely to have viral load measurements than PLWH without supporters (75.1% vs. 64.3%, p = 0.005). Table 5 shows the association between having a patient-nominated supporter and viral suppression when individuals with missing viral loads were considered “not suppressed”. Having a patient-nominated supporter was not associated with viral suppression. Having supporters who were parents or partners was associated with an increased probability of being virally suppressed in this analysis. Our sensitivity analysis that excluded PLWH with missing viral load measurements from the viral suppression denominator did not show an association between having supporters and viral suppression (aPR: 1.00, 95% CI: 0.90–1.13).

**Table 5:** Associations between having patient-nominated supporters and being virally suppressed among people living with HIV, 2011–2014.

Characteristics	Virally Suppressed (n = 790)	Not virally suppressed (n = 555)	RR (95% CI)	aRR (95% CI)
Patient-nominated supporter^a^				
Yes	717 (59.6)	485 (40.4)	1.17 (0.99–1.38)	1.18 (0.99–1.41)
No	70 (48.9)	73 (51.1)	Ref.	Ref.
Kinship of patient-nominated supporter^b^				
No supporter	73 (51.1)	70 (48.9)	Ref.	Ref.
Partner	144 (61.5)	90 (38.5)	1.21 (0.99–1.45)	1.23 (1.01–1.50)
Parent	192 (63.8)	109 (36.2)	1.25 (1.04–1.50)	1.30 (1.08–1.57)
Friend	92 (54.4)	77 (45.6)	1.07 (0.86–1.32)	1.13 (0.91–1.40)
Sibling	150 (61.2)	95 (38.8)	1.20 (0.99–1.45)	1.19 (0.98–1.44)
Second-degree relative	69 (59.5)	47 (40.5)	1.17 (0.94–1.45)	1.16 (0.93–1.45)
Offspring	25 (52.1)	23 (47.9)	1.02 (0.74–1.40)	0.89 (0.64–1.24)

All models were performed using Poisson regression with robust variances. RR = Risk Ratio, CI = Confidence Interval, aRR = adjusted Risk Ratio.

^a^Model includes the presence of a patient-nominated supporter adjusting for age, sex, sexual behavior, time since diagnosis, and AIDS at enrolment.

^b^Model includes the presence of a patient-nominated supporter disaggregated by kinship, adjusting for sex, sexual behavior, time since diagnosis, and AIDS at enrolment.

## Discussion

In this cohort study we found that most PLWH nominate their family members, partners, and friends as supporters in HIV care; and that nondisclosure of HIV status to family is associated with not having a supporter. We also found that retention in care was higher for PLWH who nominated supporters, although the association between having supporters and retention in care was only significant after two years. Our models showed that the kinship of the patient-nominated supporter appears to have a role in retention in care, as having parents or friends as supporters was associated with improved retention in care. Although we did not find an association between having supporters and viral suppression, PLWH who had supporters were more likely to have viral load measurements, indicating improved engagement.

In our setting, PLWH identify their partners, parents, siblings and friends as sources of support for HIV care, mainly among women. The kinship of supporters was similar to studies in South Africa in which patient-nominated treatment supporters were partners, mothers, sisters and friends of the PLWH [21,23]. The predominance of female supporters in both settings is supported by extensive literature that describes women as disproportionately providing health support to children, men and other women [35]. We also found differences in the proportion of supporters who were friends and partners when comparing MSM with other PLWH. Although this may represent the misclassification of same-sex partners as friends, the differential selection of supporters among MSM may also respond to different structures of social networks, social exclusion, and less stable romantic relationships [36–38].

Studies in South Africa have shown that patient-nominated supporters are selected considering supportive responses to disclosure, and availability [21,23]. We found a five-fold association between nondisclosure of HIV status to family and not having a patient-nominated supporter, in accordance with literature that supports disclosure of HIV status as a prerequisite to mobilize HIV-related social support [39–41]. Our findings show that supporters were nominated within social networks that were aware of the patient’s status, as PLWH who had not disclosed their status to their families identified supporters among their friends or partners. Having a domestic partner, being younger, and being born in Lima were also associated with having a supporter. These associations might be mediated by cohabitation with others, as these PLWH could have increased contact with partners, parents, and family members [42]. Cohabitation with others was not routinely measured in the study setting.

Previous studies have focused on the role of patient-nominated supporters in improving HIV care outcomes [21,43,44]. Navigating through the continuum of HIV care involves multiple visits to health facilities for medical and non-medical appointments, laboratory testing, and medication delivery [45,46]. In resource constrained settings, these multiple contacts with health facilities are complicated by long waiting times and competing priorities that may supersede self-care [20]. Although the specific activities that supporters perform were not studied, peers and supporters have been described to help address these barriers to retention and may provide additional navigation resources to PLWH [21,47]. The differences in retention in care by the kinship of the supporter suggest that the nature of the patient-supporter relationship might have a role in facilitating retention. Trustworthiness, good communication and availability have been identified as key factors in a successful patient-supporter relationship [21], and physical or emotional proximity to first-degree relatives or partners might lead to enhanced support. Even when social support is not enacted, perceived support may be beneficial in improving health outcomes [13,35]. Our results also suggest that the role of patient-nominated supporters in facilitating retention in care is heightened after the initial stages of care. It has been proposed that PLWH’s needs for HIV care evolve from clinical support to social support: as the PLWH achieves well-being through ART the motivation to stay in care may falter [23]. However, a previous study in our setting did not found differences in measured social support between in-care and out-of-care PLWH [48]. Whether supporters improve retention in care through instrumental, emotional or perceived dimensions of social support is still unclear and should be addressed in future studies.

Finally, our results showed higher rates of viral suppression among PLWH with supporters, though these differences were only significant when supporters were parents or partners. However, these differences were not present when PLWH without viral load measurements were excluded from analyses, and we identified an association between having viral load measurements and having supporters. We consider these results as further evidence of the facilitating role of patient-nominated supporters in engagement in HIV care. Nonetheless, we do not have sufficient evidence to affirm that patient-nominated supporters improve adherence to ART leading to reductions in viral loads. Data on self-reported treatment adherence and pharmacy refills were not available for analysis. The association between having patient-nominated supporters and these key mediators to achieve viral suppression should be assessed in future studies, particularly considering that substituting PLWH during ART refill appointments is a key role of patient-nominated supporters in the Peruvian NHP [24]. Previous studies that have evaluated the effectiveness of support provided by peers have shown moderate improvements in adherence or biological markers [49–52].

Our study has several limitations. First, more than 30% of PLWH were not evaluated by social workers and thus lacked supporter data, an unexpected finding that points towards inadequate coverage of multidisciplinary services in this setting. We assessed for selection bias comparing PLWH who were included in our analysis with those who were not. Based on our results, we consider that PLWH who engaged in baseline social work evaluations had improved overall engagement, and thus increased access to ART and retention. For this reason, the generalizability of our estimates to subpopulations with reduced engagement may be limited. Despite efforts, retrospective supporter data collection among PLWH not evaluated by social workers was not feasible. Second, the continuity of patient-nominated supporters was not measured; and both social support and disclosure of diagnosis are dynamic. However, previous studies have reported that supporters are rarely changed after nomination and that the relationship between PLWH and supporters maintains stable [21,23]. Finally, self-selection of supporters involves psychosocial factors such as stigma, personality traits, and the availability of social capital. All of these might be related to disclosure, having supporters, and patient presence in health facilities [53,54], but were not routinely assessed nor available for inclusion in our analysis. Of note, research conducted with routinely collected data remains a crucial approach towards studying the effectiveness of interventions in real-world settings: it has informed policy on the role of community treatment supporters for tuberculosis with significant advantages, including rapid answers and reduced costs [55,56].

## Conclusions

Overall, our findings indicate that patient-nominated supporters facilitate retention in HIV care and attendance to viral load measurements, but not viral suppression. Strategies that promote disclosure of HIV status to others could contribute to improved engagement in care by allowing the nomination of supporters. Interventions aimed at training supporters and enhancing the patient-supporter relationship could be developed to improve patient outcomes. Additionally, future studies could assess the feasibility, acceptability and effectiveness of strategies that support patient’s needs without warranting disclosure of HIV status and supporter nomination, such as mobile health interventions. Finally, we identified retention and suppression rates well below international recommendations. Efforts should be focused on improving these outcomes to achieve HIV epidemic control.
